# Editorial: Model organisms and experimental models: opportunities and challenges in musculoskeletal physiology

**DOI:** 10.3389/fphys.2023.1346490

**Published:** 2023-12-08

**Authors:** Matthieu Renaud, Kyu Sang Joeng, Gael Y. Rochefort

**Affiliations:** ^1^ Faculty Odontology, Tours University, Tours, France; ^2^ Department of Medicine and Bucco-Dental Surgery, Tours University Hospital, Tours, France; ^3^ N2C U1069 INSERM, Tours University, Tours, France; ^4^ Bioengineering Biomodulation and Imaging of the Orofacial Sphere, 2Bios, Tours University, Tours, France; ^5^ Department of Orthopaedic Surgery, University of Pennsylvania, Philadelphia, PA, United States; ^6^ iBrain U1253 INSEM, Tours University, Tours, France

**Keywords:** musculoskeletal physiology, model organism, experimental-animal models, arthritis, osteoporosis, denervation, tendon

Model organisms and experimental models have been widely used in the field of biosciences, including the area of musculoskeletal physiology. Several important milestones have been achieved in the field, allowing scientists to develop concepts, technologies, and methodologies to better understand physiological processes of the musculoskeletal system also in more complex living systems, including humans.

Understanding the biological, mechanical, and structural mechanisms regulating the development and homeostasis of the musculoskeletal system is essential to study pathological conditions and contributes to the development of regenerative medicine. Although model organisms cannot fully replicate human conditions and reproduce the clinical pathology of the musculoskeletal system, they play beneficial and vital roles in research for multiple reasons. They provide feasible tools to investigate the mechanism underlying disease progression, which is limited with human studies. Animal models also reproduce consistent injury models and allow genetic studies, which contribute to the understanding of cellular and molecular mechanisms regulating physiological and pathological musculoskeletal conditions (Vinnakota et al., 2016).

As part of the advanced approaches in this field, this current Research Topic of Frontiers in Physiology aims to explore the latest advances made in this topic, focusing on the many advantages of model organisms in the field of musculoskeletal physiology. This research theme also aims to shed light on the challenges and limitations that accompany their application. This current Research Topic includes four original articles and one review on this field.

The first article, entitled “*Sex differences in long-term effects of collagen-induced arthritis in middle-aged mice*” and authored by Schuh et al., focuses on rheumatoid arthritis (RA), a chronic inflammatory autoimmune disease primarily affecting synovial joints (Schuh et al.). RA is more prevalent in women. While its main features involve joint issues, it can also have extra-articular manifestations, including kidney damage and osteoporosis. The study introduces the collagen-induced arthritis (CIA) mouse model, commonly used to mimic RA in animals, with a particular emphasis on its limitations and the need for considering factors such as sex, age, and environmental conditions (Luan et al., 2021). The researchers Schuh et al. aimed to analyze the long-term effects of CIA in middle-aged male and female mice, intending to replicate the typical onset age of RA in humans. Surprisingly, the study found an earlier onset and more severe long-term effects in middle-aged male mice compared to females, contrary to the sex distribution observed in human RA. The study employed various assessments, including arthritis scoring, histological analysis, thermal imaging, and biochemical markers, to understand the dynamics and consequences of CIA. Several limitations were acknowledged, such as the lack of biochemical markers at different time points and the exclusive focus on the CIA model in mice, potentially limiting the generalizability of the findings. Despite these limitations, the study sheds light on the complexities of sex differences in the CIA model and emphasizes the importance of considering age and sex in experimental arthritis research. The conclusion highlights the need for further research to unravel the immune mechanisms underlying sex differences in RA and the CIA model. Additionally, the study encourages the adoption of novel techniques, such as infrared thermography (IRT), and emphasizes the importance of understanding the interplay between age, sex, and comorbidities in animal models of RA to enhance their clinical relevance (Schuh et al.).

In the second article, entitled “*Estradiol and zinc-doped nano hydroxyapatite as therapeutic agents in the prevention of osteoporosis; oxidative stress status, inflammation, bone turnover, bone mineral density, and histological alterations in ovariectomized rats*,” the authors Elghareeb et al. present a detailed examination of the osteoporosis-protective potential of nano zinc hydroxyapatite (ZnHA-NPs) and/or estradiol (E2) combined therapy in ovariectomized rats (Elghareeb et al.). The ovariectomy-induced osteoporosis rat model serves as a valuable and widely employed model in osteoporosis research. In this model, the removal of ovaries mimics postmenopausal conditions in females, leading to a rapid decline in estrogen levels. As estrogen plays a crucial role in maintaining bone density and preventing excessive bone turnover, its deficiency post-ovariectomy triggers bone loss, making this model particularly relevant for studying postmenopausal osteoporosis. The ovariectomy-induced osteoporosis model in rats has been instrumental in investigating potential therapeutic interventions, such as hormone replacement therapies and novel nano-biomaterials like zinc hydroxyapatite nanoparticles. The model provides insights into the mechanisms of bone turnover, hormonal regulation, and the efficacy of various treatments, contributing significantly to our understanding of osteoporosis and the development of targeted therapies (Rochefort et al., 2010; Rochefort, 2014). Osteoporosis is a significant health issue, and hormone replacement therapy (HRT) with estrogen is a common treatment but is associated with reproductive cancer risks. Hydroxyapatite nanoparticles, particularly ZnHA-NPs, are explored as an alternative remedy for osteoporosis. The study presented by Elghareeb et al. involves 35 adult female rats assigned to different treatment groups: control, ovariectomized (OVX), OVX with E2 therapy, OVX with ZnHA therapy, and OVX with both E2 and ZnHA combined therapy (Elghareeb et al.). After 3 months of treatment, various parameters, including serum bone markers, estrogen levels, oxidative/antioxidant status, inflammatory cytokines, femoral expression of specific proteins, bone mineral density, histological changes, and expression of certain markers, were assessed. The findings suggest that the combined therapy of E2 and ZnHA-NPs was more effective than individual treatments in attenuating excessive bone turnover and preventing osteoporosis. The combination treatment showed improvements in bone markers, estrogen levels, oxidative/antioxidant balance, and inflammatory cytokines. Additionally, femoral expression of specific proteins related to bone health and density, as well as histological and immunohistochemical assessments, indicated the superior efficacy of the combined therapy. The study highlights the potential of ZnHA-NPs as a nano-biomaterial for osteoporosis prevention and treatment. The combination of ZnHA-NPs and low-dose E2 showed promising results, suggesting a synergistic effect in addressing osteoporosis. The authors acknowledge certain limitations, such as the need for further exploration of ZnHA-NPs effects on non-skeletal tissues and the optimization of treatment doses and duration for improved therapeutic impact (Elghareeb et al.).

The third study, entitled “*Delayed denervation-induced muscle atrophy in Opg knockout mice*” and authored by Zhang et al., aimed to investigate the role of the osteoprotegerin/receptor activator of nuclear factor κ-B ligand/RANK (OPG/RANKL/RANK) signaling axis in muscle atrophy, particularly in the context of denervation-induced muscle damage (Zhang et al.). This OPG/RANKL/RANK signaling axis, traditionally recognized for its pivotal role in bone metabolism, has emerged as a multifaceted regulator with significant implications for muscle physiology. While extensively studied in the context of bone health, recent investigations have unveiled its intricate involvement in muscle tissues (Marcadet et al., 2022). In the present study, the research involves the use of Opg knockout mice subjected to sciatic nerve transection to induce muscle denervation (Zhang et al.). The findings challenge the existing understanding of the OPG/RANKL/RANK axis, as Opg knockout mice displayed delayed muscle atrophy and better functional recovery following denervation. The study suggests that OPG deficiency, contrary to expectations, has a protective effect on denervated muscle, as evidenced by improved functional recovery, delayed muscle atrophy, and milder activation of the ubiquitin-proteasome pathway. The protective effects of Opg knockout were associated with specific gene expression changes, including the upregulation of Inpp5k, Rbm3, and Tet2, and the downregulation of Deptor in denervated muscle. Furthermore, *in vitro* experiments using satellite cells derived from Opg knockout mice demonstrated enhanced differentiation ability compared to cells from wild-type mice. The study identifies higher expression levels of Tet2 in satellite cells from Opg knockout mice, suggesting a potential mechanistic basis for the protective effects on muscle atrophy. The results of this research expand our understanding of the OPG/RANKL/RANK axis beyond its well-established role in bone metabolism. The study highlights the complexity of this signaling pathway in different physiological and pathological conditions, emphasizing the need for context-specific investigations. The findings also have implications for potential therapeutic strategies targeting the OPG/RANKL/RANK axis in the context of muscle degeneration and atrophy, especially in conditions involving denervation (Zhang et al.).

The fourth article, entitled “*Transcriptional time course after rotator cuff repair in 6 month old female rabbits*” and authored by Vasquez-Bolanos et al., focuses on rotator cuff (RC) tears, which are particularly prevalent in individuals over the age of 60, and which represent a significant health concern, with over 400,000 surgical repairs conducted annually in the United States (Vasquez-Bolanos et al.). However, clinical studies have indicated that surgical repair does not effectively address the associated muscle atrophy and fatty infiltration observed in chronic states of the disease. This discrepancy challenges the expected anabolic response associated with muscle loading, prompting exploration into altered mechanotransduction, signaling pathways, transcriptional activity, protein synthesis, or myofibrillar assembly as potential factors. Biological activity following RC tears and surgical repairs has been extensively investigated in animal models such as mice, rats, rabbits, and sheep, each with its advantages and disadvantages. While acute RC repair models have been widely explored, there is a notable gap in understanding delayed repair scenarios, reflective of more complex human scenario. This study focuses on elucidating the time-dependent transcriptional changes in muscle after tendon repair, specifically in a chronic rabbit RC tear and repair model. Contrary to expectations, the study reveals a lack of growth or regenerative signals in the transcriptional profile after surgical repair. Instead, there are pronounced changes related to metabolism, inflammation, lipid metabolism, phagocytosis, and apoptosis. The absence of the anticipated anabolic response highlights the need for further investigation into mechanical connectivity within the muscle, mechanical signaling processes, and epigenetic changes in myocytes. This comprehensive examination of the transcriptional changes in muscle after RC repair contributes valuable insights into the biological state of the muscle post-injury and repair, paving the way for future studies to explore potential therapeutic interventions (Vasquez-Bolanos et al.).

At last, the fifth article, entitled “*Developmental origin of tendon diversity in Drosophila melanogaster*” and authored by Moucaud et al., reviewed the process of myogenesis, the development of muscle tissue, in *Drosophila* (fruit flies) and its relevance as an *in vivo* model for understanding muscle development in higher organisms (Moucaud et al.). The focus is on the myotendinous junction (MTJ), the connection between muscles and tendons, which is crucial for transmitting muscle contraction force to the skeleton. Authors emphasized the importance of tendons in the musculoskeletal system, particularly in ensuring the proper transmission of muscle forces to the skeleton. While *Drosophila* is a well-established model for studying myogenesis, the article highlights common features in the development of muscle attachment sites between vertebrates and invertebrates. Since fruit flies lack an internal skeleton, their muscles are connected to the exoskeleton through tendon-like cells. The article details various stages of tendon development in *Drosophila*, covering larval, flight, and leg muscle development. It discusses the morphological and functional diversity of tendons, emphasizing the different aspects of tendon cell specification and differentiation during embryonic development and metamorphosis. The review outlines the distinct types of tendons found in *Drosophila*, including those associated with larval muscles, flight muscles, and leg muscles. The initial cell specification of each tendon type involves the induction of the Stripe (Sr)/Egr-like transcription factor, but subsequent steps in the genetic program further distinguish tendons for specific terminal differentiation. In the concluding remarks, the article underscores the importance of tendons as precursors to connective tissues that regulate skeletal muscle differentiation, growth, and patterning. Despite their critical role, understanding connective tissue development lags behind other musculoskeletal components in vertebrates. The *Drosophila* model provides valuable insights into the diversity of muscle attachments, and recent studies have identified conserved factors contributing to tendon specification and differentiation. The article suggests that high-throughput sequencing technology could enhance understanding of the molecular mechanisms underlying tendon diversity. Additionally, the article notes the potential clinical relevance of studying tendons and muscle connective tissues, highlighting challenges such as tendon scarring and muscle dystrophies. A better understanding of connective tissue cell differentiation and its interactions with muscles and the skeleton is deemed critical for addressing mechanisms contributing to pathological conditions (Moucaud et al.).

Model organisms and experimental models play a pivotal role in advancing our understanding of musculoskeletal physiology. These models, such as mice, rats, zebrafish, and *Drosophila*, are employed in research to unravel the complexities of muscle development, bone formation, and tendon function. By studying these organisms, researchers can investigate fundamental cellular and molecular processes that are often conserved across species, providing valuable insights into human musculoskeletal physiology. These models allow for controlled experiments, genetic manipulations, and detailed observations that may not be feasible in human subjects. The information gained from model organism studies serves as a foundation for elucidating the mechanisms underlying musculoskeletal disorders, informing potential therapeutic interventions, and ultimately advancing our knowledge of the intricate workings of the musculoskeletal system.

The contributions to this Research Topic take readers on a journey to topical research activities in the specific area of model organisms and experimental models in musculoskeletal physiology. As Topic editors for this Research Topic, we are optimistic that this specific area of research will again spark inspiration and ideas for further research and development in the field.
